# Reversible Posterior Leukoencephalopathy Syndrome Induced by Pazopanib

**DOI:** 10.1186/1471-2407-12-489

**Published:** 2012-10-22

**Authors:** Leonidas Chelis, Vasilios Souftas, Kiriakos Amarantidis, Nikolaos Xenidis, Eleni Chamalidou, Prokopios Dimopoulos, Prodromos Michailidis, Evagelos Christakidis, Panagiotis Prassopoulos, Stylianos Kakolyris

**Affiliations:** 1 Department of Medical Oncology, University General Hospital of Alexandroupolis, Dragana, 68100, Alexandroupolis, Thrace, Greece; 2 Department of Radiology and Medical Imaging, Democritus University of Thrace, Alexandroupolis, Greece

**Keywords:** Reversible posterior leukoencephalopathy syndrome, Pazopanib, Renal cell carcinoma

## Abstract

**Background:**

The reversible posterior leukoencephalopathy syndrome is a clinical/radiological syndrome characterized by headache, seizures, impaired vision, acute hypertension, and typical magnetic resonance imaging findings. There are several reports in the literature that depict its occurrence in cancer patients. The list of common anticancer and supportive care drugs that predispose to reversible posterior leukoencephalopathy syndrome is expanding and includes not only a large number of chemotherapeutic agents but also an increased number of new targeted drugs, particularly angiogenesis inhibitors such as bevacizumab,sorefenib and sunitinib. Pazopanib is an oral tyrosine kinase inhibitor targeting vascular endothelial growth factor receptor, platelet-derived growth factor receptor, and c-Kit which after a positive phase III randomized clinical trial in patients with advanced renal cell cancer received FDA approval for the treatment of advanced renal cell carcinoma. Until now no cases of reversible posterior leukoencephalopathy syndrome induced by pazopanib have been reported.

**Case report:**

We present the case of a 40 years old female patient with heavily pre-treated metastatic renal cell carcinoma who received pazopanib as salvage treatment. After 21 days of pazopanib therapy the patient referred to the emergency department with epileptic seizure, impaired vision at both eyes and headache. MRI of the brain revealed subcortical oedema at the occipital and parietal lobes bilaterally. She was treated with anticonvulsants, i.v. administration of mannitol and antihypertensives and she recovered completely from her symptoms and was discharged on the tenth hospital day. A brain MRI performed 3 weeks after showed that the subcortical oedema had been subsided.

**Conclusion:**

In conclusion this is the first case of pazopanib induced reversible posterior leukoencephalopathy syndrome. Although usually reversible, this syndrome is a serious and potentially life threatening adverse effect, if untreated, that should be considered by physicians treating metastatic renal cell carcinoma patients with pazopanib.

## Background

The reversible posterior leukoencephalopathy syndrome (RPLS) has been reported as a distinct clinical entity by several investigators [1,2]. It is a clinical/radiological syndrome characterized by headache, seizures, impaired vision, acute hypertension, and typical magnetic resonance imaging findings such as hyperintensity on T2-weighted and fluid-attenuated inversion recovery (FLAIR) images involving the posterior circulation areas, especially the parietal and occipital lobes which are usually symmetric. The hallmark of RPLS is the almost complete recovery in the majority of the cases of clinical symptoms and brain imaging findings, which usually occurs within days to weeks [3].

The correlation of the syndrome with several conditions like hypertensive encephalopathy, eclampsia, collagen vascular disorders, renal dysfunction, thrombotic thrombocytopenic purpura, acute porphyria, Guillain-Barré syndrome and the usage of immunosuppressive agents (especially calcineurin inhibitors like cyclosporine) is well defined [3]. There are numerous case reports in the literature that depict its occurrence in cancer patients. The list of common anticancer and supportive care drugs that predispose to RPLS is expanding and includes a large number of chemotherapeutic agents such as cisplatin, cyclophosphamide, high-dose corticosteroids, L-asparaginase, and growth supportive factors such as granulocyte colony-stimulating factor (G-CSF), and erythropoietin [4]. In the last few years an increased number of case reports involving new targeted drugs, particularly angiogenesis inhibitors have been reported. Agents such as bevacizumab, sunitinib and sorafenib and other targeted drugs have been implicated in new cases of RPLS [5-10].

Pazopanib is an oral tyrosine kinase inhibitor (TKI) targeting vascular endothelial growth factor receptor (VEGFR), platelet-derived growth factor receptor (PDGFR), and c-Kit [11]. After a positive phase III randomized clinical trial in patients with advanced renal cell cancer [12] pazopanib was approved by the US FDA in October 2009 for the treatment of advanced renal cell carcinoma (RCC). The toxicity profile of the drug mimics that of other anti-angiogenesis TKIs consisting mainly of diarrhoea, hypertension, nausea and fatigue but until now no cases of RPLS induced by pazopanib have been reported [13,14].

### Case report

We present the case of a 40 years old female patient who at the age of 30 underwent right nephrectomy for a clear cell renal cell carcinoma. Five years later she developed lung and osseous metastases and the biopsy of the lung lesion revealed metastatic RCC. Bevacizumab plus interferon-A was administered as 1^st^ line treatment and the disease evaluation after three months revealed disease progression. She was treated with sunitinib malate as 2^nd^ line therapy for one year achieving stable disease as best response. At relapse, sorafenib was given as 3^rd^ line treatment and the patient achieved a remarkable partial response lasting almost for 36 months. Due to disease progression everolimus was initiated as 4^th^ line treatment and the disease evaluation after 6 months revealed progression at her bone metastases, stable disease at lung lesions and no evidence of brain metastases. The ECOG performance status of the patient was 1 due to mild pain at her right hip, she had no history of hypertension and since pazopanib has shown effectiveness also in cytokine-pretreated patients with metastatic RCC [12], we decided to administer pazopanib as 5^th^ line treatment. Her medical history was insignificant for other diseases than metastatic renal carcinoma. The concomitant medication during pazopanib treatment consisted of fentanyl transdermal at 25mg and tramadol hydrochloride.

**Figure 1:**
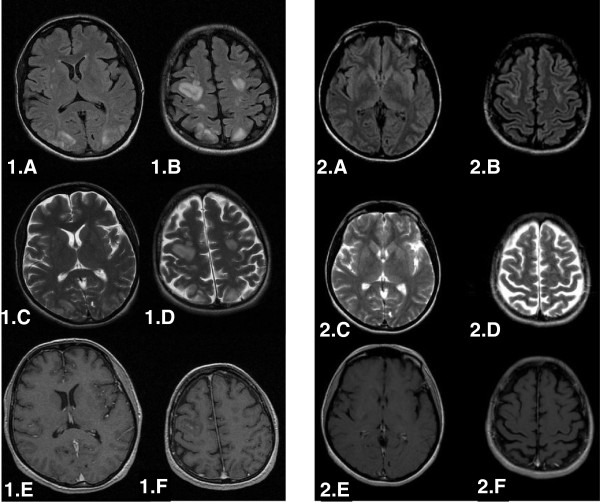
**Brain MRI at the onset of the syndrome (1) and three weeks later (2).:** Subcortical oedema at the occipital and parietal lobes bilaterally shown as hyperintense signals on FLAIR and T2 sequences, absence of lesions on T1 sequence. 1.**A**, 1.**B** (FLAIR), 1.**C**, 1.**D** (T2), 1.**E**, 1.**F** (T1), respectively. Three weeks later the subcortical oedema had been subsided from both FLAIR and T2 sequences. : 2.**A**, 2.**B**, 2.**C**, 2.**D** respectively.

After 21 days of pazopanib therapy the patient referred to the emergency department with epileptic seizure, impaired vision at both eyes and bilateral, temporal and pulsutive headache. The physical examination revealed hypertension 165/105 mmHg (grade III toxicity), suspension of both direct and indirect papillary reflexes for both eyes and bilateral blurred vision and diplopia. On neurological examination there was no evidence of lateralizing or focal features. Blood tests, including CBC, blood chemistry, coagulation study and gasometry, were unremarkable. The performed urine analysis showed no evidence of significant proteinuria (dipstick staining: 1+) . Retinoscopy was also insignificant. MRI of the brain revealed subcortical oedema at the occipital and parietal lobes bilaterally (hyperintense signals on FLAIR and T2 sequences, absence of lesions on T1 sequence). These findings are given in Figure. 1: 1.A, 1.B (FLAIR), 1.C, 1.D (T2), 1.E, 1.F (T1), respectively.

Pazopanib administration was withheld and the patient was treated with anticonvulsants (phenytoin), i.v. administration of mannitol and antihypertensives : furosemide 40 mg given i.v on the first date and perindopril 4 mg given orally the following days. One day later the seizures were stopped, her visual ability was restored and the blood pressure was normalized after few hours with furosemide and remained within normal levels the next days. The headache was more intense the first day but after the normalization of the blood pressure it was gradually improved and completely remitted after 5 days. A lumbar puncture was performed and the cerebrospinal fluid analysis revealed no cells, normal physical characteristics, normal chemical tests and negative microscopic examination. In addition the viruses tests for HSV1, HSV2, CMV, EBV were negative. She completely recovered from her symptoms and was discharged on the tenth hospital day.

The patient underwent a brain CT scan, two weeks after the onset of the symptoms, which was negative for abnormal findings. A brain MRI performed 3 weeks after confirmed the CT scan findings showing that the subcortical oedema had been subsided from both FLAIR and T2 sequences. (Figure. 1: 2.A, 2.B, 2.C, 2.D). Due to the serious nature of the adverse drug reaction re-administration of pazopanib was not carried out and the patient was re-challenged with sorafenib because of the excellent past response. Unfortunately, the patient died 2 months later because of disease progression in the lungs with no signs of central nervous disease.

Causality assessment carried out using the World Health Organization-Uppsala Monitoring Centre criteria revealed that the reaction had ‘probable/likely’ relationship with pazopanib usage [15]. The association was also evaluated using the Naranjo algorithm for estimating the probability of adverse drug reactions and a score of 6 indicated a ‘probable’ relationship [16].

## Discussion

The case described above matches both clinical and radiological criteria of RPLS and to the best of our knowledge this is the first case of pazopanib induced RPLS. There are several other neurological complications such as brain metastasis, stroke or, drug-induced delirium which can be clinically confused with RPLS, and therefore, the differential diagnosis of the syndrome is very important; brain MRI is mandatory for the correct diagnosis.

The treatment for RPLS is symptomatic, similar to malignant hypertension, with the goal of lowering diastolic blood pressures to around100 mm Hg within few hours. Even if hypertension is mild, it is still recommended to reduce blood pressure [4]. Whereas treatment with anticonvulsants is the standard of care for seizures associated with the acute phase of RPLS, this does not necessitate longterm antiepileptic treatment [3]. In cases of RPLS induced by anticancer drugs, it is recommended that the offending agent be withdrawn, if possible, and that blood pressure be controlled [4]. In selected cases where there is no alternative treatment, anti-VEGF agents can be re-administered at lower dose with close monitoring of blood pressure [17].

Although the pathogenesis of RPLS is not fully understood there are several mechanisms that could be proposed to explain how the syndrome was triggered by pazopanib in our patient. The administration of pazopanib for mRCC patients has been frequently correlated with hypertension ranging between 40-93% and a significant proportion of them (9-25%) developed severe (grade III) hypertension [12,18]. Even a modest increase in blood pressure, if it is acute, especially in the presence of an underlying endothelial dysfunction, may exceed protective autoregulatory mechanisms resulting in breakdown of the blood–brain-barrier, pathological vasodilation, capillary leakage which result to extravasation of fluids into the brain parenchyma [19]. The posterior circulation is more vulnerable to those sudden changes of blood pressure due to the relative lack of adrenergic nerves around pial and intracerebral vessels in comparison with the anterior circulation of the brain [20]. Moreover, pazopanib has strong antiangiogenic properties via inhibition of the VEGF and PDGF pathways in vessels cells which suggests that the vascular endothelium may be an important site of toxicity [11].Disrupted or immature vascular endothelium may result in vascular leak and white matter oedema characteristic of RPLS especially in the setting of a sudden increase of the blood pressure like that occurring in our patient.

It is worth mentioning that in a study about the association of RPLS with anticancer drugs [21], a predominance of RPLS cases affecting women, especially those of premenopausal age was reported. There are several genes that are oestrogen-regulated and are actively involved in the vascular physiology like endothelin-1, prostacyclin cyclooxygenase, VEGF and endothelial nitric oxide synthase (eNOS). Our patient was a 40 years old premenopausal woman and oestrogen activation of those genes may also contributed to endothelial toxicity and played a role in the pathogenesis of RPLS [22,23]. This hypothesis is also fueled by the well established correlation of RPLS and eclampsia.

## Conclusion

In conclusion this is the first case of pazopanib induced RPLS. Although usually reversible, RPLS is a serious and potentially life threatening adverse effect, if untreated, that should be considered by physicians treating mRCC patients with pazopanib. As we have entered the era of multiple antiangiogenic targeted agents for the treatment of mRCC more cases of RLPS are anticipated.

### Consent

Written informed consent was obtained from the patient’s husband for publication of this Case report and any accompanying images. A copy of the written consent is available for review by the Series Editor of this journal.

## Abbreviations

RPLS: Reversible posterior leukoencephalopathy syndrome; FLAIR: Fluid-attenuated inversion recovery; G-CSF: Granulocyte colony-stimulating factor; TKI: Tyrosine kinase inhibitor; VEGFR: Vascular endothelial growth factor receptor; PDGFR: Platelet-derived growth factor receptor; RCC: Renal cell carcinoma; eNOS: Endothelial nitric oxide synthase.

## Competing interests

The authors declare that they have no competing interests.

## Authors' contributions

CL: conception and design of the manuscript, manuscript drafting. SV: acquisition, analysis and interpretation of data. AK: data acquisition and critical revision of the manuscript. XN: data acquisition and critical revision of the manuscript. CE: coordination and helped to draft the manuscript. DP: data acquisition and analysis, review of the literature. MP: data acquisition and analysis, review of the literature. CE: data acquisition and analysis, review of the literature. PP: critical revision and final approval of the manuscript. KS: critical revision and final approval of the manuscript. All authors read and approved the final manuscript.

## Pre-publication history

The pre-publication history for this paper can be accessed here:

http://www.biomedcentral.com/1471-2407/12/489/prepub
